# Caustic Ingestion in the Elderly: Influence of Age on Clinical Outcome

**DOI:** 10.3390/molecules22101726

**Published:** 2017-10-14

**Authors:** Blazena Caganova, Tatiana Foltanova, Erik Puchon, Elena Ondriasova, Silvia Plackova, Tomas Fazekas, Magdalena Kuzelova

**Affiliations:** 1 Department of Occupational Medicine and Toxicology, National Toxicological Information Centre, University Hospital Bratislava, Bratislava 83305, Slovak; caganova@ntic.sk (B.C.); plackova@ntic.sk (S.P.); 2 Department of Pharmacology and Toxicology, Faculty of Pharmacy, Comenius University in Bratislava, Bratislava 81499, Slovak; puchone@gmail.com (E.P.); ondriasova@fpharm.uniba.sk (E.O.); kuzelova@fpharm.uniba.sk (M.K.); 3 Department of Physical Chemistry of Drugs, Faculty of Pharmacy, Comenius University in Bratislava, Bratislava 81499, Slovak; fazekas@fpharm.uniba.sk

**Keywords:** acids, hydrochloric acid, caustic poisoning, corrosive substance, elderly, respiratory complications, mortality rate

## Abstract

Caustic poisonings are still associated with many fatalities. Studies focusing on the elderly are rare. The purpose of the present study was to compare the clinical outcomes of caustic ingestion injury in elderly and non-elderly adults with regard to gender, intent of exposure, substance ingested, severity of mucosal injury, complications, and mortality. Caustic substance exposures reported to the National Toxicological Information Centre in Slovakia during 1998–2015 were reviewed retrospectively. The patients were divided into two groups: the non-elderly (<60 years) and elderly adults (≥60 years). The mortality rate in the elderly was significantly higher (elderly 23.0% vs. non-elderly 11.3%; *p* = 0.041). The risk of fatal outcome in the elderly was increased by acid ingestion (OR = 7.822; *p* = 0.002), particularly hydrochloric acid (OR = 5.714, *p* = 0.006). The incidence of respiratory complications was almost two times higher in the elderly was 31.1% vs. 17.4% for the non-elderly (*p* = 0.037). Respiratory complications significantly correlated with an increased mortality rate (*p* = 0.001) in the elderly whereas there was no association between GI complications and mortality in the elderly (*p* = 0.480). Elderly patients with respiratory complications had the poorest clinical outcomes. The highest risk of complications and fatalities was observed in patients after hydrochloric acid ingestion.

## 1. Introduction

Caustic poisonings pose a serious problem for healthcare providers due to high morbidity and mortality. Chemicals and chemical products stored in homes are the source of many accidental or intentional exposures that can be seen in people of different ages [1]. Caustic ingestion contribute to frequent causes of inquiries to the National Toxicological Information Centre (NTIC) in Slovakia [2].

Diluted acids or alkalis usually induce limited mucosal damage. In contrast, concentrated caustic agents with pH <2 or >12 can result in severe esophageal damage with either colliquative (alkali) or coagulative necrosis (acids) and, at the same time, a wide range of gastrointestinal injuries which can lead to late post-corrosive complications [3,4,5,6].

From the toxicological point of view, pH often fails to predict the extent of injury after exposure and reliance on pH alone might result in clinical errors in patient management. The determined pH value must be taken into account with other physical-chemical parameters, e.g., viscosity, physical form, type, and concentration of consequential ingredients, amount ingested, contact time, and premorbid state of the esophagus, which may contribute to the outcome of poisoning. No marker exists to accurately predict a product’s potential for injury [1,7].

Most patients with mild injuries recover without serious consequences but a large number of post-corrosive poisonings result in serious chemical injuries with complications and a high mortality rate [5,8,9]. Acute ingestions of corrosive substances are very often followed by complications, such as pneumonia, respiratory failure, bleeding, perforation, strictures, and fistula [7,10]. Mortality is most often caused by tracheal necrosis and perforation of the esophagus or stomach, followed by mediastinitis or peritonitis [8,11,12].

Elderly people are especially vulnerable to intoxication. Since they are more fragile than younger age groups, poisoning leads to more severe complications. According to the European Network for Safety among the Elderly accidental poisoning with chemicals is the third leading cause of death by unintentional injury in the elderly population [13,14].

The European population is ageing. There has been a rapid increase in the number of elderly people worldwide, and there are more old people nowadays than at any time in history. The proportion of the population aged 60 and over is also growing each year. According to the World Health Organization (WHO) by the year 2025, the world will have host 1.2 billion people aged 60 and over, rising to 1.9 billion in 2050 [15,16].

Studies on the elderly group of people are in general rare and there is only little research focusing on the caustic poisonings in the elderly. The purpose of the present retrospective study was to compare the clinical outcomes of caustic ingestion injury in elderly and non-elderly adults with regard to gender, intent of exposure, substance ingested, severity of mucosal injury, complications, and mortality.

## 2. Results

### 2.1. Demographic and Caustic Injury Description

We retrospectively analyzed medical reports of 176 adult patients with acute corrosive ingestion who underwent an endoscopic evaluation within 24 h after ingestion.

The non-elderly group was comprised of 115 (65.3%) patients younger than 60 years whereas in the elderly group there were 61 (34.7%) patients 60 years of age or older. The mean age for all the patients was 51.8 (19–87) years.

Males were more than twice as likely to be exposed to caustic substances compared to females (*p* = 0.030). Most of the exposures were accidental (*n* = 108; 61.4%). Suicidal ingestion occurred in approximately one third of the patients (*n* = 63; 35.8%). In the elderly group, the difference between accidental and suicidal exposure was only 6.5% (50.8 versus 44.3%). Nonetheless, no significant differences regarding the gender and circumstances were observed between the non-elderly and elderly groups.

Demographic characteristics of the two groups are presented in Table 1.

Together, 164 of all 176 patients (93.2%) had positive endoscopic findings. According to the Zargar’s classification [17] grade I was present in 51 (29.0%) patients, grades IIa and IIb in 74 (42.3%), and severe injury of grades IIIa and IIIb were found in 39 cases (22.2%). No significant differences regarding the severity of mucosal damage or the location of caustic injury were observed between the non-elderly and elderly groups.

### 2.2. Chemical Origin of the Caustic Substances and Their Corrosive Potential

The most frequently ingested group of caustic chemicals were acids, alkalis, and bleaches, followed by cationic detergents, paraquat/diquat, and glyphosate. The most often ingested chemical substance was sodium hydroxide, followed by hydrochloric acid and chlorine-based bleach (Table 2).

The significant differences regarding the type of ingested substance were observed between the two groups. The exposures to acids were almost two times more common in the elderly group (OR = 1.927 (1.005–3.695); *p* = 0.047). In hydrochloric acid these exposures were even more frequent (OR = 3.128 (1.295–7.551); *p* = 0.009). On the contrary, the exposures to alkalis prevailed in the non-elderly group (OR = 0.430 (0.196–0.943); *p* = 0.032).

**Figure 1:**
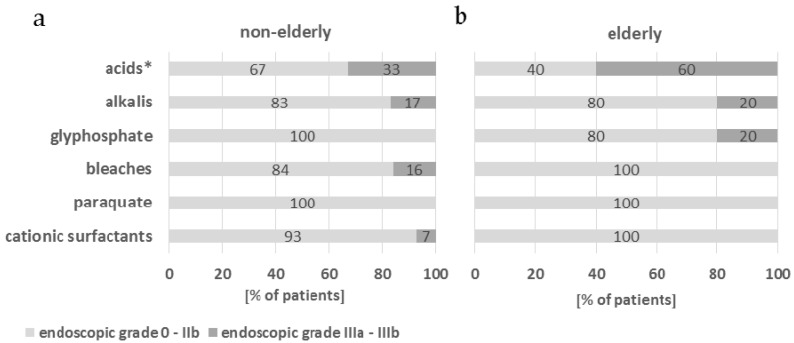
Severity of the caustic injury related to the chemical origin of the substance—comparison of elderly and non-elderly group. (**a**) Non-elderly group, *n* = 115; (**b**) elderly group, *n* = 61. Cochran’s and Mantel Haenszel statistics, * *p* < 0.001.

Related to the chemical origin of the substance, severe caustic injury (IIIa–IIIb) was most frequently present in patients after ingestion of acids. This finding was especially apparent in elderly patients (*p* = 0.001) (Figure 1).

### 2.3. Complications and Clinical Outcome

Table 3 illustrates presence of selected complications that influenced caustic injury mortality. Thirty-nine patients (22.2%) suffered from respiratory complications during their hospital stay. Aspiration pneumonia was diagnosed in 14 patients (8.0%). Assisted ventilation due to respiratory distress was required in 35 patients (19.9%). The overall incidence of respiratory complications was almost two times higher in the elderly (31.1%) than in non-elderly group (17.4%; *p* = 0.037). Patients in the elderly group developed respiratory failure (27.9%) more often compared to younger adults (15.7%; *p* = 0.050).

Thirty-eight patients (21.6%) developed gastrointestinal complications, mostly GI-bleeding (13.6%), perforation (8.5%), and peritonitis/mediastinitis (8.0%). Two patients (1.1%) developed oesophago-tracheal fistulas. Early stricture formation (within three weeks after caustic ingestion) was observed in two patients (1.1%). The incidence mediastinitis/peritonitis was higher in the elderly (13.1%) compared to non-elderly group (5.2%). Nonetheless, there was no significant difference in the incidence of GI complications between the two groups.

Leukocytosis developed in 18.2% of all patients, more frequently in the elderly group (34.4 vs. 9.6%; *p* = 0.001). Antibiotics were administered to 54.0% of all patients. Antibiotic usage was significantly more frequent in the elderly group compared to younger adults (63.9% vs. 48.7%; *p* = 0.050%).

Together, 149 of all 176 patients (84.7%) were admitted to the hospital due to caustic injury. The mean length of hospital stay was 6.0 days (1–45). The difference between the non-elderly and elderly groups was significant (4.8 vs. 8.2 days; *p* = 0.003) as presented in Table 3.

The chemical nature of the corrosive substance was related to the incidence of complications. The significant risk of complications was observed in elderly after ingestion of acids particularly hydrochloric acid (Table 4, OR = 10.694 (2.546–44.919); *p* = 0.001). Twenty-seven of the patients died due to the severity of their injury and complications. In our study, the mortality rate was 15.3%. The mortality rate in the elderly was significantly higher (23.0%) compared to the non-elderly group (11.3%; *p* = 0.041). The data are presented in Table 3. Causes of death were ingestion of strong acids (*n* = 22), paraquat (*n* = 3), and glyphosate (*n* = 2) as presented in Table 5.

The risk of fatal outcome in elderly was significantly increased by acid ingestion (OR = 7.822 (1.898–32.241); *p* = 0.002), particularly hydrochloric acid (OR = 5.714, (1.527–21.391); *p* = 0.006), as presented in Table 6.

According to results of univariate analysis the mortality risk was significantly higher in elderly patients compared to the non-elderly age group. The multivariate binary logistic regression identified that respiratory complications significantly correlated with increased mortality rate (*p* = 0.001) in the elderly group, whereas there was no association between GI complications and mortality in the elderly (*p* = 0.480). The quality of new predictive logistic model was verified by evaluation of the respective confusion matrix, where 9.8% were false positives and 1.6% were false negatives.

## 3. Discussion

Caustic injury remains an important medical problem worldwide despite various educational and regulatory efforts to reduce its occurrence. In the years 1998–2015 the National Toxicological Information Centre (NTIC) in Bratislava registered 1847 caustic substance exposures from all over Slovakia. These exposures accounted for 3.8% from the total number of registered inquiries. In total, 318 patients underwent an early endoscopy—176 adults and 142 children.

Particularly serious were these intoxications in elderly adults. Caustic poisoning in the elderly is a significant problem. The majority of cases is unintentional and may result from improper use of the product, improper storage, mistaken identification, confusion, or dementia. Depression is also common in the elderly and suicide attempts are more likely to be successful in this age group. The loneliness and social isolation of some elderly people are a contributing reason for late presentation to the hospital after intoxication [14,18]. The most serious poisonings are mostly related to suicidal attempts when a large amount of a caustic product is swallowed and the damage is usually extensive [3,10,11,19]. In the present study the incidence of suicidal attempts was higher in elderly (44.3%) compared to the non-elderly group (31.3%).

Intoxications with caustic agents produce numerous and severe post-corrosive complications of the upper gastrointestinal tract, and may also cause injuries of the respiratory system [5]. A recent study by World Society of Emergency Surgery showed that 10% of the patients sustaining caustic injury experienced immediate gastrointestinal (GI) complication including perforation and bleeding [9]. Approximately 5–15% developed respiratory distress and required ventilatory support [7,9]. The incidence of respiratory complications was significantly higher in the elderly compared to the non-elderly group. Respiratory failure was present two times more often in the group of elderly patients compared to younger patients. This corresponds with the study by Chang et al. [20] where incidence of respiratory failure was almost four times higher in the elderly group than in younger adults. There was no significant difference regarding the severity of mucosal injury between the non-elderly and elderly group. In addition, the incidence of GI complications, such as bleeding, perforation, stricture, and fistula formation was also independent of age. Respiratory complications significantly correlated with increased mortality rate (*p* = 0.037) in the elderly group, whereas there was no association between GI complications and mortality in the elderly (*p* = 0.948). Use of antibiotics was more frequent in the elderly group (63.9%) compared to 48.7% in younger adults. It is difficult to say if higher usage of antibiotics was caused by caustic agent intoxication or there is a need for the higher use of antibiotics in elderly patients in general. On the other hand, we confirmed that respiratory complications and leukocytosis were present more often in the elderly than in the non-elderly group, which could explain the higher use of antibiotics. As expected, the length of hospital stay was significantly higher in the population of elderly patients, with mean value 8.2 days compared to 4.8 days for the non-elderly group.

Mortality after caustic ingestion is high worldwide and ranges from 5% to 20% [3,5,7,9,21,22]. In our study the mortality rate was 15.3%. Twenty-seven of the patients died due to the severity of their injury and complications. Causes of death were ingestion of strong acids, paraquat, and glyphosate. The mortality in elderly was two times higher compared to younger adult group. This corresponds with the study by Chang where mortality rate was almost three times higher in the elderly than in the group of non-elderly patients [19,20].

Although there are no uniform guidelines for the nutritional support of patients after caustic injury and the approach varies from patient to patient, appropriate nutritional support reduces risk of malnutrition or infection. This might be of big importance particularly in the elderly patients [23,24].

The chemical nature of the corrosive substance was significantly related to the severity of complications and mortality rate.

The ingestion of common bleach (sodium hypochlorite or hydrogen peroxide) is rarely associated with severe damage to the upper GI-tract [25]. In the present study ingestion of bleach was associated with low risk of injury. After intentional ingestion of household bleach, only three of 28 patients (younger than 60 years) had a severe degree of burns (grade IIIa). The ingestion of cationic surfactants caused a mild degree of burns. None resulted in severe injury or death. Glyphosate is considered a mild caustic agent that usually produces esophageal injuries of grades I, IIa, and IIb [26]. Severe (grade III) lesions may ensue following substantial ingestions with gastrointestinal hemorrhage [27]. We recorded two fatal cases after glyphosate ingestion in elderly group. A 70-year-old man died after ingestion of 200 mL of glyphosate and 69-year old woman suffered from IIIb glyphosate injury followed by perforation which had resulted in death after 45 days after ingestion. Paraquat is a relatively mild caustic agent, but with high risk of mortality. The endoscopic examination is not routine in the paraquat intoxication management, although it may result in perforation, mediastinitis, and pneumomediastinum [28]. In our study eight patients ingested paraquat or diquat. However, three of them died (one patient from the elderly group) after serious paraquat burns with severe systemic complications. The sale, advertisement, and supply of paraquat products have been banned since July 2008 in the European Union [27,29,30]. Some studies found the outcome for patients ingesting alkalis to be more favorable than for those ingesting acids with regard to mucosal injury, perforation, systemic complications, and mortality [3,4]. Our results also showed that ingestion of strong alkalis was associated with a lower risk of fatal outcome.

Intoxications with acids prevailed over intoxications with alkalis in the whole group of patients and especially in elderly. Acids, in contrast to alkali substances, have a poor taste and are more irritating, which may lead to a patient’s choking. This may predispose the patient to aspirate the caustic material, with subsequent airway compromise [31]. In our study the majority of complications were observed in patients after acid ingestion. Several studies have also reported less favorable outcome for acid ingestion [6,7,32,33,34,35,36]. Hydrochloric acid caused severe complications in the whole group of patients and, especially, severe complications were present in the elderly group. Hydrochloric acid is a widespread industrial product used in numerous countries as a rust remover, pool cleaner, drain and toilet bowl cleaner, and in other household products [30,34,37]. Severe, even fatal, intoxications were reported from several European countries [6,33,34,35,36]. According to the European chemical agency (ECHA) legislation there are no restrictions in registration or use of hydrochloric acid-containing products in EU [38]. Hydrochloric acid is easily available in Slovakia. People can buy concentrated solution practically in unlimited volume for a minimal price. Additionally, it is a part of cleaning products used in the household, too. Hydrochloric acid was often misused in suicidal attempts where severe damage could be expected but serious intoxications were present even though intoxication was accidental. We registered 14 fatal cases after hydrochloric acid ingestion. In the elderly group the risk of fatal outcome was almost six times higher after hydrochloric acid ingestion. The widespread availability of highly caustic household products has increased the number of intoxications in the population. Prevention of caustic ingestion is essential, as the tissue injury occurs within a few minutes.

## 4. Materials and Methods

We retrospectively reviewed all caustic substance exposures that were the subject of inquiries to the National Toxicological Information Centre (NTIC) in Slovakia between January 1998 and December 2015. NTIC is the only toxicological centre in the country and serves a population of about 5.5 million inhabitants [39]. In the study we included only adult patients (>18 years) with corrosive ingestions who underwent urgent endoscopic evaluation within 24 h and whose medical reports were forwarded to the NTIC. The patients were divided into two groups according to age: non-elderly (<60 years) and elderly adults (≥60 years) [15]. The parameters examined and compared in these two groups involved gender, circumstance of exposure (accidental or suicidal), type of ingested agent, degree of mucosal injury, associated complications, and length of hospital stay. Endoscopic findings were classified according to the Zargar’s classification (Table 7) depending on the depth and extent of burns as 0, I, IIa, IIb, IIIa, and IIIb [17].

Any complications during hospitalization were recorded. We focused on gastrointestinal (GI) and respiratory disorders. Recorded upper GI complications included bleeding, perforation, stricture, and fistula formation. Bleeding was defined as melena, hematemesis, and/or coffee-ground vomitus. Perforation was diagnosed by the presence of free air on a plain chest radiograph. Stricture was defined as dysphagia, symptoms of regurgitation, or difficulty in swallowing with confirmation by endoscopy, and/or upper GI radiography [3,5]. Only strictures developed within three weeks post-ingestion were recorded. Recorded respiratory complications were pneumonia and respiratory distress with requirement for ventilatory support [26]. Additionally, we recorded the presence of leukocytosis and use of antibiotics.

Information on all ingested corrosive substances was sought and this was supplemented with material safety datasheet details. Chemicals with corrosive effects were divided into eight categories according to their use and chemical characteristics. In addition to the two major groups, acids and alkalis, we also included bleaches (chlorine or oxygen-based), cationic surfactants, and caustic herbicides (glyphosate and paraquat/diquat).

### 4.1. Main Characteristics of Chemical Substances Involved

Alkali agents with pH levels greater than 12 are highly corrosive and it is widely documented that they cause liquefactive burns and necrosis [27,40].

Acids may cause blistering ulceration and penetrating necrosis. Coagulation burns may develop with thee formation of an eschar. In general, the lower the pH of an acid, the more corrosive it is. Substances with a pH level less than 2 are considered highly corrosive [27,40].

Chlorine-based bleaches contain chlorine or hypochlorite; the pH is usually 12–13. They are used as common disinfectants and bleaching agents. Bleaches with a chorine/hypochlorite concentration of 10% or greater are considered corrosive. There have been cases where bleaches with lower hypochlorite concentrations have led to serious effects when ingested in large amounts [27,41].

Oxygen-based bleaches (e.g., hydrogen peroxide, H_2_O_2_) are colorless, acidic oxidizing agents available in a variety of concentrations from 3 to 90%. They are used in numerous household products, including disinfectants, chlorine-free bleaches, fabric stain removers, and hair dyes. Concentrations above 10% are potentially corrosive [27].

Cationic surfactants (usually quaternary ammonium compounds, e.g., *N*-alkyl-*N*-benzyl-*N*,*N*-dimethylammonium chloride) are corrosive depending on the concentration. They are used as general disinfectants. Concentrations above 7.5% cause corrosive damage to the upper gastrointestinal tract [27].

Paraquat (1,1’-dimethyl-4,4’-bipyridylium dichloride) and diquat (1,1’-ethylene-2,2’-bipyridilium dibromide) are non-selective bipyridyl herbicides whose toxic effects are similar. Concentrated solutions (>20%) may cause severe corrosive injury when ingested. Both are extremely toxic and poisoning may be fatal in humans.

Glyphosate (*N*-(phosphonomethyl) glycin) is a non-selective weedkiller. Preparations containing glyphosate are usually formulated as the isopropylamine salt of glyphosate in various concentrations in aqueous solutions containing surfactant. Larger amounts may cause corrosive effects [27].

Other than the caustic agents that were described above, involved, e.g., combinations of different chemicals, unidentified acid or alkali-based products or caustic drugs (salicylic acid in keratolytic concentration, potassium permanganate).

### 4.2. Statistical Analysis

Data were entered into MS Excel and transferred into a Statistical Package for Social Sciences for Windows 19.0 (SPSS 19.0). Continuous variables are expressed as mean within range. Categorical variables are presented as frequencies and percentages. Pearson’s chi square test and Fisher’s exact test (for *n* < 5) were used for the evaluation of the association between binary categorical variables. To describe the consistent difference in proportions (severity of injury, chemical origin of the corrosive agent) across ages we used Cochran’s and Mantel Haenszel statistics. Logistic regression was used to describe the relationship between mortality, complications (gastrointestinal and/or respiratory), and chemical origin of the corrosives, demographic characteristics, and causes of the caustic ingestion, respectively. The calculated binary logistic model was used to predict mortality. The extent of misclassified items, as the respective confusion matrix, was calculated and evaluated with contingency analysis. Differences at *p* ≤ 0.05 were considered statistically significant and clinical significance was further evaluated, if applicable.

## 5. Conclusions

Our 18-year study confirmed that mortality after caustic ingestion is significantly higher in the elderly compared to younger adults. Elderly patients with respiratory complications have the poorest clinical outcomes. Gastrointestinal complications appear to have no impact on the mortality in the elderly. The highest risk of complications and fatalities was observed in patients after ingestion of acids. Hydrochloric acid caused the highest number of serious intoxications in the whole group of patients. This finding is especially apparent in elderly patients, and will present a serious threat as long as widespread availability of highly caustic household products will be unrestricted. As the tissue injury occurs within a few minutes, prevention of caustic ingestion is essential.

## Figures and Tables

**Table 1:** Patient’s demographic and caustic injury parameters.

Variables	Total (*n* = 176)	Non-Elderly (<60 years, *n* = 115)	Elderly (≥60 years, *n* = 61)
Mean age (years)	51.8 (19–87)	43.0 (19–59)	68.8 (60–87)
**Gender**			
Male (%)	124 (70.5)	82 (71.3)	42 (68.9)
Female (%)	52 (29.5)	33 (28.7)	19 (31.1)
**Circumstance**			
Accident (%)	108 (61.4)	77 (67.0)	31 (50.8)
Intention (%)	63 (35.8)	36 (31.3)	27 (44.3)
Missing (%)	5 (2.8)	2 (1.7)	3 (4.9)
**Endoscopic grade**			
0 (%)	6 (3.4)	4 (3.5)	2 (3.3)
I (%)	51 (29.0)	34 (29.6)	17 (27.9)
IIa (%)	65 (36.9)	47 (40.9)	18 (29.5)
IIb (%)	9 (5.1)	6 (5.2)	3 (4.9)
IIIa (%)	19 (10.8)	10 (8.7)	9 (14.8)
IIIb (%)	20 (11.4)	11 (9.6)	9 (14.8)
Missing (%) ^1^	6 (3.4)	3 (2.6)	3 (4.9)
**Location of caustic injury**			
Oropharyng (%)	103 (58.5)	70 (60.9)	33 (54.1)
Oesophagus (%)	98 (55.7)	59 (51.3)	39 (63.9)
Stomach (%)	98 (55.7)	63 (54.8)	35 (57.4)
Duodenum (%)	33 (18.8)	20 (17.4)	13 (21.3)

^1^ In six cases with fatal outcome the endoscopic examination could not be completed due to high risk of perforation.

**Table 2:** Chemical origin of caustic substances frequently involved.

Caustic Substance	Total (%) *n* = 176	Non-Elderly (<60 years, *n* = 115)	Elderly (≥ 60 years, *n* = 61)	*p*
Acids (%)	58 (33.0)	32 (27.8)	26 (42.6)	0.047
HCl (%)	24 (13.6)	10 (8.7)	14 (23.0)	0.009
H_2_SO_4_ (%)	13 (7.4)	6 (5.2)	7 (11.5)	0.131
H_3_PO_4_ (%)	6 (3.4)	4 (3.5)	2 (3.3)	1.000
HNO_3_ (%)	2 (1.1)	2 (1.7)	0 (0.0)	0.544
Alkalis (%)	46 (26.1)	36 (31.3)	10 (16.4)	0.032
NaOH (%)	25 (14.2)	20 (17.4)	5 (8.2)	0.096
Na_2_SiO_3_ (%)	9 (5.1)	6 (5.2)	3 (4.9)	1.000
KOH (%)	4 (2.3)	3 (2.6)	1 (1.6)	1.000
Bleaches (%)	28 (15.9)	19 (16.5)	9 (14.8)	0.760
Chlorine-based bleaches (%)	20 (11.4)	14 (12.2)	6 (9.8)	0.642
Oxygen-based bleaches (%)	8 (4.5)	5 (4.3)	3 (4.9)	1.000
Cationic surfactants (%)	20 (11.4)	15 (13.0)	5 (8.2)	0.335
Glyphosate ^1^ (%)	9 (5.1)	4 (3.5)	5 (8.2)	0.279
Paraquat ^2^, Diquat ^3^ (%)	8 (4.5)	5 (4.3)	3 (4.9)	1.000
Others (%)	7 (4.0)	4 (3.5)	3 (4.9)	0.695

^1^ Glyphosate—(*N*-(phosphonomethyl) glycin), ^2^ Paraquat—(1,1’-dimethyl-4,4’-bipyridylium dichloride), ^3^ Diquat—(1,1’-ethylene-2,2’-bipyridilium dibromide). Pearson’s χ^2^, Fischer’s exact test for *n* < 5, *p* ≤ 0.05.

**Table 3:** Incidence of complications and mortality rate according to age.

Parameter	Total (*n* = 176)	Non-Elderly (≤60 years, *n* = 115)	Elderly (>60 years, *n* = 61)	*p*
Respiratory complications (%)	39 (22.2)	20 (17.4)	19 (31.1)	0.037
Pneumonia (%)	14 (8.0)	7 (6.1)	7 (11.5)	0.209
RF ^1^ (%)	35 (19.9)	18 (15.7)	17 (27.9)	0.050
GI ^2^ complications (%)	38 (21.6)	25 (21.7)	13 (21.3)	0.948
Bleeding (%)	24 (13.6)	18 (15.7)	6 (9.8)	0.285
Perforation (%)	15 (8.5)	9 (7.8)	6 (9.8)	0.649
Peritonitis/Mediastinitis (%)	14 (8.0)	6 (5.2)	8 (13.1)	0.065
Fistula (%)	2 (1.1)	0 (0.0)	2 (3.3)	0.119
Stricture (%)	2 (1.1)	2 (1.7)	0 (0.0)	0.300
Leukocytosis (%)	32 (18.2)	11 (9.6)	21 (34.4)	0.001
Antibiotic usage (%)	95 (54.0)	56 (48.7)	39 (63.9)	0.050
Mean length of hospital stay (%)	6.0 (1–45) (*n* = 149)	4.8 (1–22) (*n* = 97)	8.2 (1–45) (*n* = 52)	0.003
Mortality (%)	27 (15.3)	13 (11.3)	14 (23.0)	0.041

^1^ RF—respiratory failure, ^2^ GI—gastrointestinal. Pearson’s χ^2^ test, Fischer’s exact test for *n* < 5, *p* ≤ 0.05.

**Table 4:** Relative rate of complications (GI/respiratory) in elderly according to the chemical origin of the chemical substance.

Caustic Substance	OR	95% CI	*p*
Acids (%)	9.130	2.766–30.128	0.001
HCl (%)	10.694	2.546–44.919	0.001
Alkalis (%)	0.664	0.154–2.874	0.729
NaOH (%)	1.111	0.171–7.203	1.000
Glyphosate (%)	1.111	0.171–7.203	1.000
Paraquat (%)	0.818	0.070–9.561	1.000

OR—odds ratio, 95 %CI—95% confidence interval. Pearson’s χ^2^ test, Fischer’s exact test for *n* < 5, *p* ≤ 0.05.

**Table 5:** Caustic substances which caused fatal outcomes.

Caustic Substance	Fatal Outcome (*n* = 176)	Non-Elderly (≤60 years, *n* = 115)	Elderly (>60 years, *n* = 61)
Acids (%)	22 (12.5)	11 (9.6)	11 (18.0)
HCl (%)	14 (8.0)	7 (6.1)	7 (11.5)
H_2_SO_4_ (%)	4 (2.3)	0 (0.0)	4 (6.6)
HNO_3_ (%)	2 (1.1)	2 (1.7)	0 (0.0)
Unidentified acid (%)	2 (1.1)	2 (1.7)	0 (0.0)
Glyphosate (%)	2 (1.1)	0 (0.0)	2 (3.3)
Paraquat (%)	3 (1.7)	2 (1.7)	1 (1.6)
Total (%)	27 (15.3)	13 (11.3)	14 (23.0)

Pearson’s χ^2^ test, Fischer’s exact test for *n* < 5, *p* ≤ 0.05.

**Table 6:** Relative rate of fatal outcome in the elderly according to the chemical origin of the toxic substance.

Caustic Substance	OR	95% CI	*p*
Acid (%)	7.822	1.898–32.241	0.002
HCl (%)	5.714	1.527–21.391	0.006
Glyphosate (%)	2.444	0.366–16.337	0.322
Paraquat (%)	1.731	0.145–20.637	0.549

OR–odds ratio, 95% CI–95% confidence interval. Pearson’s χ^2^ test, Fischer’s exact test for *n* < 5, *p* ≤ 0.05.

**Table 7:** Zargar’s grading classification of mucosal injury caused by ingestion of caustic substances [17].

Grade 0	Normal examination
Grade I	Edema and hyperemia of the mucosa
Grade IIa	Superficial ulceration, erosions, friability, blisters, exudates, hemorrhages, whitish membranes
Grade IIb	Grade IIa plus deep discrete or circumferential ulcerations
Grade IIIa	Small scattered areas of multiple ulceration and areas of necrosis with brown-black or greyish discoloration
Grade IIIb	Extensive necrosis
